# A Neuroergonomics Approach to Mental Workload, Engagement and Human Performance

**DOI:** 10.3389/fnins.2020.00268

**Published:** 2020-04-07

**Authors:** Frédéric Dehais, Alex Lafont, Raphaëlle Roy, Stephen Fairclough

**Affiliations:** ^1^ISAE-SUPAERO, Université de Toulouse, Toulouse, France; ^2^School of Biomedical Engineering, Science and Health Systems, Drexel University, Philadelphia, PA, United States; ^3^School of Psychology, Liverpool John Moores University, Liverpool, United Kingdom

**Keywords:** neuroergonomics, performance prediction, degraded attentional and executive mental states, task engagement, mental workload

## Abstract

The assessment and prediction of cognitive performance is a key issue for any discipline concerned with human operators in the context of safety-critical behavior. Most of the research has focused on the measurement of mental workload but this construct remains difficult to operationalize despite decades of research on the topic. Recent advances in Neuroergonomics have expanded our understanding of neurocognitive processes across different operational domains. We provide a framework to disentangle those neural mechanisms that underpin the relationship between task demand, arousal, mental workload and human performance. This approach advocates targeting those specific mental states that precede a reduction of performance efficacy. A number of undesirable neurocognitive states (mind wandering, effort withdrawal, perseveration, inattentional phenomena) are identified and mapped within a two-dimensional conceptual space encompassing task engagement and arousal. We argue that monitoring the prefrontal cortex and its deactivation can index a generic shift from a nominal operational state to an impaired one where performance is likely to degrade. Neurophysiological, physiological and behavioral markers that specifically account for these states are identified. We then propose a typology of neuroadaptive countermeasures to mitigate these undesirable mental states.

## Introduction

A study of mental workload is fundamental to understanding the intrinsic limitations of the human information processing system. This area of research is also crucial for investigation of complex teaming relationships especially when interaction with technology necessitates multitasking or a degree of cognitive complexity.

### The Growth of Mental Workload

Mental workload has a long association with human factors research into safety-critical performance (Moray, 1979; O’Donnell and Eggemeier, 1986; Hancock and Meshkati, 1988; Hancock and Desmond, 2001; Wickens and Tsang, 2014; Young et al., 2015). Forty years have passed since the publication of the seminal collection edited by Moray (1979) and the study of mental workload remains an active topic in contemporary human factors research; a keyword search based on Google Scholar listed more than 200,000 articles published on the topic since 2000, see also Table 1 in Young et al. (2015). The significance of human mental workload for those technological trends that are forecast during the second machine age (Brynjolfsson and McAfee, 2014) guarantees its importance for human factors research in future decades.

The lineage of mental workload incorporates a number of theoretical perspectives, some of which precede the formalization of the concept itself. Early work linking physiological activation to the prediction of performance (Yerkes and Dodson, 1908; Duffy, 1962) was formalized into an energetical model of attentional resources (Kahneman, 1973) that emphasized a dynamic relationship between finite information processing capacity and variable cognitive demands (Norman and Bobrow, 1975; Navon and Gopher, 1979; Wickens, 1980). The descriptive quality of the early work on attentional resources was sharpened by cognitive models of control (Broadbent, 1971; Schneider et al., 1984; Shallice and Burgess, 1993). Hybrid frameworks that place cognitive processes within a resource framework have been hugely influential in the field, such as the multiple resource model (Wickens, 1984, 2002, 2008; Wickens and Liu, 1988) whereas others introduced agentic features, such as dynamic self-regulation and adaptation, within models of human performance (Hockey et al., 1986; Hockey, 1997). For instance, Hancock and Warm (1989)’s dynamic adaptive theory (DAT) postulates that the brain seeks resource homeostasis and cognitive comfort. However, environmental stressors can progressively shift individual’s adaptive abilities from stability to instability depending on one’s cognitive and psychological resources. The DAT is an extension of the Yerkes and Dodson inverted-U law, in a sense that very low (hypostress) and very high (hyperstress) task demands can both degrade the adaptability and consequently impair performance. All these perspectives are united by a characterization of the human information processing system as a finite resource with limited capacity (Kramer and Spinks, 1991).

### Mental Workload Measurement

Research into the measurement of mental workload has outstripped the development of theoretical frameworks. Measures of mental workload can be categorized as performance-based, or linked to the process of subjective self-assessment, or associated with psychophysiology or neurophysiology. Each category has specific strengths and weaknesses (O’Donnell and Eggemeier, 1986; Wierwille and Eggemeier, 1993) and the sensitivity of each measurement type can vary depending on the level of workload experienced by the operator (De Waard, 1996). The development of multidimensional measures led inevitably to an inclusive framework for mental workload. The cost of this integration is dissociation between different measures of mental workload, e.g., Yeh and Wickens (1988), and an integrated workload concept that remains poorly defined from a psychometric perspective (Matthews et al., 2015).

There are a number of reasons that explain why mental workload is easy to quantify but difficult to operationalize. The absence of a unified framework for human mental workload, its antecedents, processes and measures has generated a highly abstract concept, loosely operationalized and supported by a growing database of inconsistent findings (Van Acker et al., 2018). The absence of a general explanatory model is complicated by the fact that mental workload, like stress and fatigue (Matthews, 2002), is a transactional concept representing an interaction between the capacities of the individual and the specific demands of a particular task. Within this transactional framework, mental workload represents a confluence between inter-individual sources of trait variability (e.g., skill, IQ, personality), intra-individual variation (e.g., emotional states, motivation, fatigue), and the specific configuration of the task under investigation (see also Table 2 in Van Acker et al., 2018).

For the discipline of human factors, the study of mental workload serves two primary functions: (a) to quantify the transaction between operators and a range of task demands or technological systems or operational protocols, and (b) to predict the probability of performance impairment during operational scenarios, which may be safety-critical. One challenge facing the field is delineating a consistent relationship between mental workload measurement and performance quality on the basis of complex interactions between the person and the task. The second challenge pertains to the legacy and utility of limited capacity of resources as a framework for understanding those interactions.

In the following sections, we detail some limitations of mental resources and advocate the adoption of a neuroergonomic approach (Sarter and Sarter, 2003; Parasuraman and Rizzo, 2008; Parasuraman and Wilson, 2008; Mehta and Parasuraman, 2013; Ayaz and Dehais, 2018) for the study of mental workload and human performance. The neuroergonomic framework emphasizes a shift from limited cognitive resources to characterizing impaired human performance and associated states with respect to neurobiological mechanisms.

### Toward a Limit of the Theory of Limited Resources

The concept of resources represents a foundational challenge to the development of a unified framework for mental workload and prediction of human performance. The conception of a limited capacity for information processing is an intuitive one and has been embedded within several successful models, e.g., multiple resources (Wickens, 2002). But this notion has always been problematic because resources are a general-purpose metaphor with limited explanatory powers (Navon, 1984) that incorporate both cognitive processes (e.g., attention, memory) and energetical constructs (e.g., mental effort) in ways that are difficult to delineate or operationalize. The allegorical basis of resources almost guarantees an abstract level of explanation (Van Acker et al., 2018) that is accompanied by divergent (Matthews et al., 2015), and sometimes contradictory operationalizations (Yeh and Wickens, 1988; Annett, 2002).

For example, the theory of limited cognitive resources predicts that exposure to task demands that are sustained and demanding can impair performance due to resource depletion via self-regulation mechanisms at the neuron-level (i.e., local-sleep state theory, see Van Dongen et al., 2011) or compromise access to resources mechanisms (Borragan Pedraz and Peigneux, 2016). However, this type of explanation fails to clarify why non-challenging tasks, such as passive monitoring (Matthews et al., 2002, 2010) can promote episodes of mind wandering whereby attention drifts from task-related to task-irrelevant thoughts (Smallwood et al., 2008; Durantin et al., 2015; Smallwood and Schooler, 2015). Although some propositions, such as the theory of “malleable resources” (Young and Stanton, 2002), have intuited this paradox, this theory is at a highly descriptive level and remains difficult to operationalize.

Similarly, the occurrence of stressful and unexpected operational scenarios is known to impair executive functioning and provoke perseveration, see Dehais et al. (2019) for review. Perseveration is defined as a tendency to continue an action after cessation of the original stimulation, which is no longer relevant to the goal at hand (Sandson and Albert, 1984). For example, several studies conducted on emergency evacuation situations reported irrational and perseverative behaviors even when tasks were simple and undemanding (Proulx, 2001; Kobes et al., 2010). A paradigmatic situation is the one in which people fail to escape from fire because they push the door instead of pulling it. Perseveration can also have devastating consequences during safety-critical tasks, such as aviation (O’Hare and Smitheram, 1995; Orasanu et al., 1998; Reynal et al., 2017) and in the medical domain (Bromiley, 2008). This category of performance impairment cannot be explained solely through the prism of limited mental resources. Operators who persist with an erroneous strategy, such as an aircrew who attempt to land their craft at all costs despite bad weather conditions, are generally capable of performing the required actions and tend to invest greater effort even as their task goal becomes difficult or even impossible to achieve (Dehais et al., 2010, 2012).

The concept of limited cognitive resources could explain failures of attention such as inattentional blindness (Brand-D’Abrescia and Lavie, 2008) or deafness (Raveh and Lavie, 2015). Both categories describe an inability to detect unexpected stimuli, such as alarms from the interface (Dehais et al., 2011, 2014), and represent breakdown of selective attention due to the presence of competing demands on the human information processing system. It has been demonstrated that individuals with greater information processing capacity (i.e., higher working memory span) exhibit superior ability with respect to divided and sustained attention (Colflesh and Conway, 2007; Unsworth and Engle, 2007), and therefore, should be less susceptible to the effects of inattention during the performance of demanding tasks. However, this hypothesis is contradicted by the absence of any correlation between individual differences in processing capacity and the occurrence of inattentional blindness (Bredemeier and Simons, 2012; Beanland and Chan, 2016; Kreitz et al., 2016a) or deafness (Kreitz et al., 2016b; Dehais et al., 2019).

This research suggests that the limited resource model cannot account for critical lapses of attention and executive functioning that are observed under conditions of high mental workload. Therefore, we must go beyond the limitations of the resource concept as an explanatory model of mental workload and turn our attention to the neural underpinnings of attention and behavior (Parasuraman et al., 1999).

## Resources: a Neuroergonomic Perspective

The last three decades have witnessed a revolution in our understanding of neural mechanisms that are fundamental to attention and human performance. Progress in the field has been driven by the development of advanced and portable neuroimaging techniques, which permit non-invasive examination of the “brain at work.” Neuroergonomics is a multidisciplinary field born from these technical innovations that is broadly defined as the study of the human brain in relation to performance at work and in everyday settings (Parasuraman and Rizzo, 2008). The goal of this field is to integrate both theories and principles from ergonomics, neuroscience and human factors in order to provide insights into the relationship between brain function and behavioral outcomes in the context of work and everyday life (Rizzo et al., 2007; Parasuraman and Rizzo, 2008; Parasuraman and Wilson, 2008; Lees et al., 2010; Ayaz and Dehais, 2018).

### The Multiple Biological Substrates of Mental Resources

The incorporation of neurophysiological measures of mental workload offers a reductive pathway to the reification of resources and those neurobiological states associated with impaired performance. At a fundamental level, the functioning of neurons within the brain is a form of limited resource (Beatty, 1986), requiring oxygen and glycose to generate cellular energy in the form of adenosine triphosphate (ATP) while having a very limited capacity to store these energy substrates (Saravini, 1999). The same logic holds for ions (e.g., potassium, calcium, sodium) that play a key role in nerve impulses. It is also reasonable to consider neural networks as resources with respect to their supporting glial cells (e.g., astrocytes), which ensure the processing of information (Mandrick et al., 2016). Understanding the interactions between neurobiological resources with reference to fundamental processes in brain physiology represents a crucial approach within neuroergonomic analysis of mental workload (Parasuraman and Rizzo, 2008; Ayaz and Dehais, 2018).

### Brain and Inhibitory Mechanisms

The brain must be considered to be a “noisy” organ, whereby assembly of neurons are constantly responsive to environmental stimulations, see Pandemonium architecture as an early example, such as Selfridge (1959). Inhibitory mechanisms are implemented to cancel out cerebral noise by mitigating the activation of distracting neuronal assemblies (Polich, 2007). This process may occur at a local level via lateral inhibition, whereby groups of neurons can attenuate the activity of their neighbors in order to be “better heard” (Coultrip et al., 1992). The same mechanism can also take place via top-down regulation, known as inhibitory control, wherein high-level cortical areas (e.g., prefrontal cortex) reduce task- or stimulus-irrelevant neural activities (Munakata et al., 2011). However, these inhibitory mechanisms can also curtail the capacity of the brain to consider new or alternative information, thus leading to perseveration (Dehais et al., 2019). An appropriate metaphor is to consider a group led by an authoritarian leader who is totally engaged with one specific goal or strategy and does not listen to alternative viewpoints of other members of the group. Within this metaphor, information processing resources are present (i.e., group members) but are disregarded in the presence of an overriding directive (i.e., the leader). In other words, high mental workload leads to impaired performance, not because of limited resources *per se*, but because of those neurological mechanisms designed to prioritize a specific goal or directive.

### The Non-linear Effects of Neuromodulation

**FIGURE 1:**
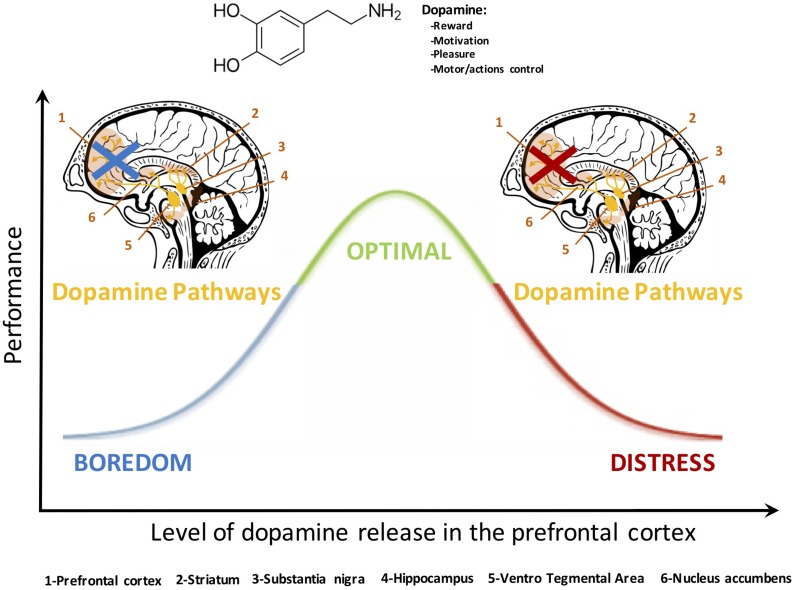
The dopamine pathway exerts a quadratic control over the PFC. A low or a high release of this neurochemical depresses PFC activation whereas an adequate concentration ensures optimal executive functioning (Vijayraghavan et al., 2007; Robbins and Arnsten, 2009). These neurobiological considerations bring interesting highlights to understand the mechanisms underlying the Yerkes and Dodson inverted-U law and the dynamic adaptability theory (Hancock and Warm, 1989). They also provide a relevant prospect to relate motivational aspects to behavioral responses. The noradrenaline pathway mediates the PFC activity and executive functioning in a similar fashion (see Aston-Jones and Cohen, 2005).

The prefrontal cortex (PFC) is a brain structure often identified as the neurophysiological source of limited resources (Posner and Petersen, 1990; Parasuraman, 2003; Ramsey et al., 2004; Modi et al., 2017). The PFC serves a control function during routine cognitive operations, such as: action selection, retrieval/updating in working memory, monitoring and inhibition (Ramnani and Owen, 2004; Ridderinkhof et al., 2004). It is often activated during high levels of cognitive demand (Ayaz et al., 2012; Herff et al., 2014; Racz et al., 2017; Gateau et al., 2018; Fairclough et al., 2019) and dysfunction of this structure is known to degrade performance (Sandson and Albert, 1984; Dolcos and McCarthy, 2006). However, the PFC is complex and its function is subject to the quadratic influence of neuromodulation via the influence of noradrenaline and dopamine (Arnsten, 2009; Arnsten et al., 2012). Noradrenaline is associated with the mediation of arousal (Chrousos, 2009) whereas dopamine is involved in the processing of reward with regard to the ongoing tasks (Schultz, 2002). Both catecholamines exert an inverted-U relationship with the PFC neurons (Vijayraghavan et al., 2007; Robbins and Arnsten, 2009), a reduction of these neurochemicals will depress the firing rate of noradrenergic and dopaminergic PFC neurons (see Figure 1). This mechanism may explain why unstimulating and non-rewarding tasks (e.g., passive supervisory control over a sustained period) can inhibit executive functioning and induce mind wandering. Conversely, excessive levels can also have a deleterious effect by suppressing PFC neuron firing rate (Birnbaum et al., 1999). In addition to decreasing the activity of the PFC, dopamine and noradrenaline activate subcortical areas, such as basal ganglia, that trigger automated schemes and initiate automatic responses (Wickens et al., 2007). These automated behaviors have an advantage of speed compared to flexible but slower behaviors generated by the prefrontal cortex (Dolan, 2002). This neurological switch from prefrontal to subcortical areas, is presumed to derive from the early age of humanity to ensure survival (Arnsten, 2009). In modern times, it manifests itself as a process of defaulting to well-learned behaviors, which are effective for only operational situations that are simple and familiar. This is the mechanism that promotes perseveration (Dehais et al., 2019) in task scenarios that are complex and novel (Staal, 2004; Ellenbogen et al., 2006) or offer intrinsic, short-term rewards, e.g., landing at all costs after a long transatlantic flight (Causse et al., 2013). These fundamental neurological mechanisms illustrate that impaired operational performance cannot be simply explained in terms of limited resources, such as a concentration of dopamine, but must be viewed from a neuroergonomic perspective that emphasizes the complexity of interactions between brain areas that evolved over thousands of years.

### Attentional Dynamics and Dominance Effects

The existence of information processing resources can also be conceptualized as functional attentional networks in the brain. Michael Posner was the first to pioneer a network approach to the operationalization of resources in the early days of neuroimaging (Posner and Tudela, 1997). His influential analysis (Posner and Petersen, 1990; Posner and Dehaene, 1994; Petersen and Posner, 2012; Posner, 2012) described how specific networks were dedicated to the particular functions of attentional regulation, e.g., alerting, orientation, focus. This conceptualization developed into the delineation of a dorsal fronto-parietal network (e.g., intraparietal cortex, superior frontal cortex) that supports focused attention on specific task-relevant stimuli and a corresponding ventral fronto-parietal network (e.g., temporo-parietal cortex, inferior frontal cortex) in the right hemisphere, which activates in a bottom-up fashion to reorientate attention to interruptive stimuli (Corbetta and Shulman, 2002; Corbetta et al., 2008). Under nominal conditions, interaction between the dorsal and the ventral pathways ensure optimal trade-off between those attentional strategies associated with exploitation and exploration. However, under conditions of high task demand or stress or fatigue, this mechanism may become biased toward dominance of the dorsal over the ventral network, leading to attentional phenomena associated with inflexibility (Todd et al., 2005; Durantin et al., 2017; Edworthy et al., 2018; Dehais et al., 2019a). A similar dynamic of bias and dominance is apparent in the relationship between the dorsal and ventral pathways and the default mode network (Andrews-Hanna et al., 2014), which is associated with mind-wandering, spontaneous thoughts and disengagement from task-related stimuli (Fox et al., 2015).

### Performance Monitoring and Effort Withdrawal

The capacity of the brain to monitor performance quality and progress toward task goals is another important function of the PFC during operational performance. The posterior medial frontal cortex (pMFC) is a central hub in a wider network devoted to performance monitoring, action selection and adaptive behavior (Ullsperger et al., 2014; Ninomiya et al., 2018). The pMFC is sensitive to error and failure to achieve a task goal (Ullsperger et al., 2007); the detection of failure represents an important cue for compensatory strategies, such as increased investment of mental effort (Hockey, 1997). This network is particularly important when the level of task demand experienced by the operator is associated with a high rate of error and increased probability of failure. The model of motivational intensity (Richter et al., 2016) predicts that effort is withdrawn from task performance if success likelihood is appraised to be very low (Hopstaken et al., 2015); similarly, models of behavioral self-regulation (Carver and Scheier, 2000) argue that task goals can be adjusted downward (i.e., lower levels of performance are tolerated as acceptable) or even abandoned if goal attainment is perceived to be impossible. There is evidence that increased likelihood of failure is associated with deactivation of the PFC (Durantin et al., 2014; Ewing et al., 2016; Fairclough et al., 2019), for operational performance where failure can often jeopardize the safety of oneself and others, increased likelihood of failure can also provoke strong emotional responses that are associated with stress and cognitive interference (Sarason et al., 1990), which can function as distractors from task activity in their own right (Dolcos and McCarthy, 2006; Qin et al., 2009; Gärtner et al., 2014).

This neuroergonomic approach provides a biological basis upon which to develop a concept of limited human information processing, with respect to competing neurological mechanisms, the influence of neuromodulation in the prefrontal cortex and antagonist directives between different functional networks in the brain. The prominence of inhibitory control coupled with competition between these neural networks delineate a different category of performance limitations during extremes of low vs. high mental workload, i.e., simultaneous activation of functional networks with biases toward mutually exclusive stimuli (external vs. internal) or contradictory directives (focal attention vs. reorientation of attention).

## Understanding Performance Related Mental States

**FIGURE 2:**
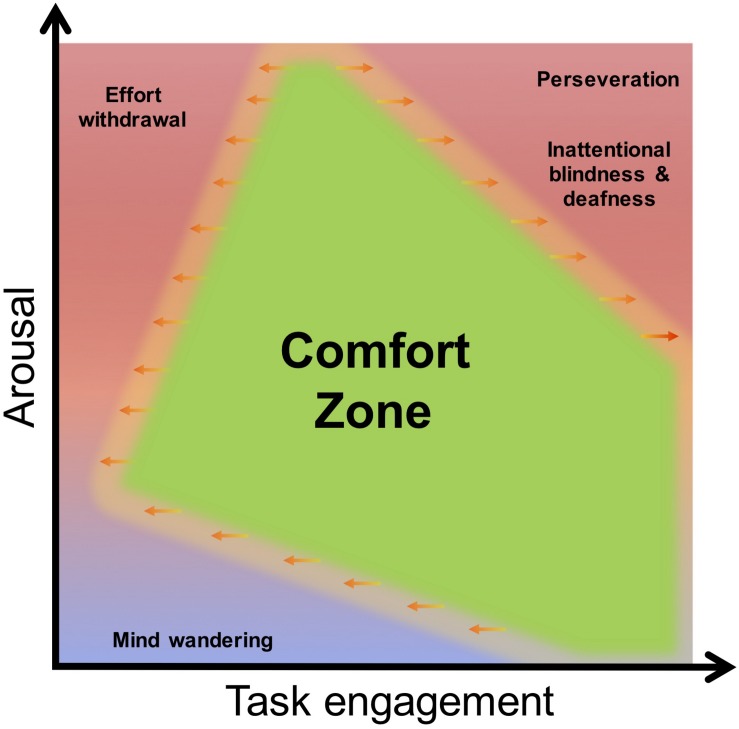
Performance, arousal and task engagement: the green zone conceptually describes the operator’s “comfort zone” where performance is optimal. The degraded mental states are mapped across a “task engagement” axis and an “arousal” axis. Interestingly, this point of view makes it possible to link the notion of engagement and degraded behavior in a simple way.

The previous sections have highlighted the complexity of those brain dynamics and networks that can introduce inherent limitations on human information processing. On the basis of this analysis, it is reasonable to target neurophysiological states and their associated mechanisms that account for impaired human performance (see Prinzel, 2002). This review has identified a number of suboptimal neurocognitive states that are predictive of degraded performance such as: mind wandering, effort withdrawal, perseveration, inattentional blindness and deafness. These states may be conceptually mapped along orthogonal dimensions of task engagement and arousal (Figure 2). Engagement is defined as an effortful investment in the service of task/cognitive goals (Pope et al., 1995; Matthews et al., 2002; Stephens et al., 2018), whereas arousal represents a state of physiological readiness to respond to external contingencies (Pribram and McGuinness, 1975).

### The Transactional Dimensions of Engagement and Arousal

The rationale for considering the dimension of task engagement is that performance is driven by goals and motivation (Bedny and Karwowski, 2004; Fairclough et al., 2013; Leontiev, 2014). Goal-oriented cognition theorists argue for the existence of mechanisms dedicated to maintain engagement (Atkinson and Cartwright, 1964), which are associated with an activation of an executive (Baddeley and Hitch, 1974) or task-positive network (Harrivel et al., 2013) within which the dorsolateral prefrontal cortex (DLPFC) exerts a crucial role (Goldman-Rakic, 1987; Curtis and D’Esposito, 2003). This structure plays a key role in the maintenance and updating of information that is relevant for ongoing task performance. The same structure interacts with dorsal and ventral attentional pathways to shift and focus attention to the most relevant stream of task-related information (Johnson and Zatorre, 2006). It is argued that human performance can be assessed in the context of a continuum of task engagement, ranging from disengagement (effort withdrawal, mind wandering) to high-engagement (perseveration, inattentional phenomena Lee, 2014).

**FIGURE 3:**
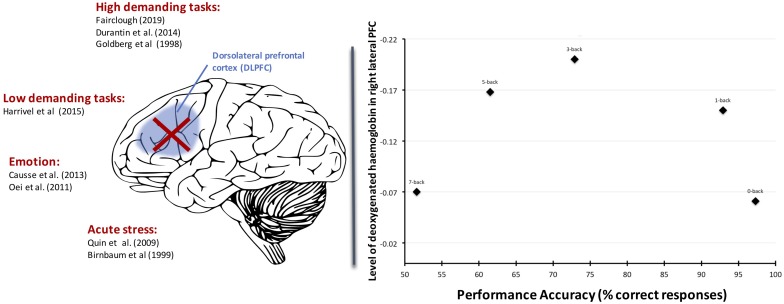
Left part: Several types of stressors can yield to the deactivation of the DLPFC and in return drastically induce collapse of performance. Right part: An illustration with the N-Back task: the right-DLPFC deactivates when the task demands exceed mental capacity (7-Back condition) and is associated with reduced performance efficacy and effort withdrawal (from Fairclough et al., 2019).

Arousal makes an important contribution to the conceptual space illustrated in Figure 2 because it modulates the homeostasis of the executive (see Arnsten, 2009 for a review) and attentional networks (see Coull, 1998 and Aston-Jones and Cohen, 2005 for review) via the dopaminergic and noradrenergic pathways. For instance, both extremes of low (Harrivel et al., 2013; Durantin et al., 2015) and high arousal can disengage the DLPFC (Goldberg et al., 1998; Arnsten, 2009; Qin et al., 2009; Causse et al., 2013; Durantin et al., 2014; Fairclough et al., 2019) and impair performance (see Figure 3 for summary). Similarly, low (Dehais et al., 2018) and high levels of arousal (Hancock and Warm, 1989; Tracy et al., 2000; Pecher et al., 2011) can alter the interactions between the dorsal and ventral attentional networks and indistinctly that lead either to inattentional phenomena (Molloy et al., 2015; Todd et al., 2005) or effort withdrawal (Oei et al., 2012; Dehais et al., 2015).

### Monitoring Performance Through Degraded Mental States

Table 1 presents a mapping between extremes of high and low engagement and arousal, their related neurocognitive states and how these states may be operationalized using neurophysiological measures in the laboratory and the field. Monitoring the activation and deactivation of the DLPFC represents a promising generic avenue to predict impaired performance across diverse states such as: mind wandering (Christoff et al., 2009; Harrivel et al., 2013), effort withdrawal (Ayaz et al., 2007; Izzetoglu et al., 2007; Durantin et al., 2014; Modi et al., 2018; Fairclough et al., 2018, 2019) and perseveration (Dehais et al., 2019). However, other neurological networks and sites should be considered as part of this analysis. Mind wandering is characterized by the concomitant activation of the default network, which includes the median prefrontal cortex (Christoff et al., 2009; Harrivel et al., 2013) and areas of the parietal cortex (Christoff et al., 2009).

**TABLE 1:** Psycho-physiological and behavioral markers of different mental states related to engagement.

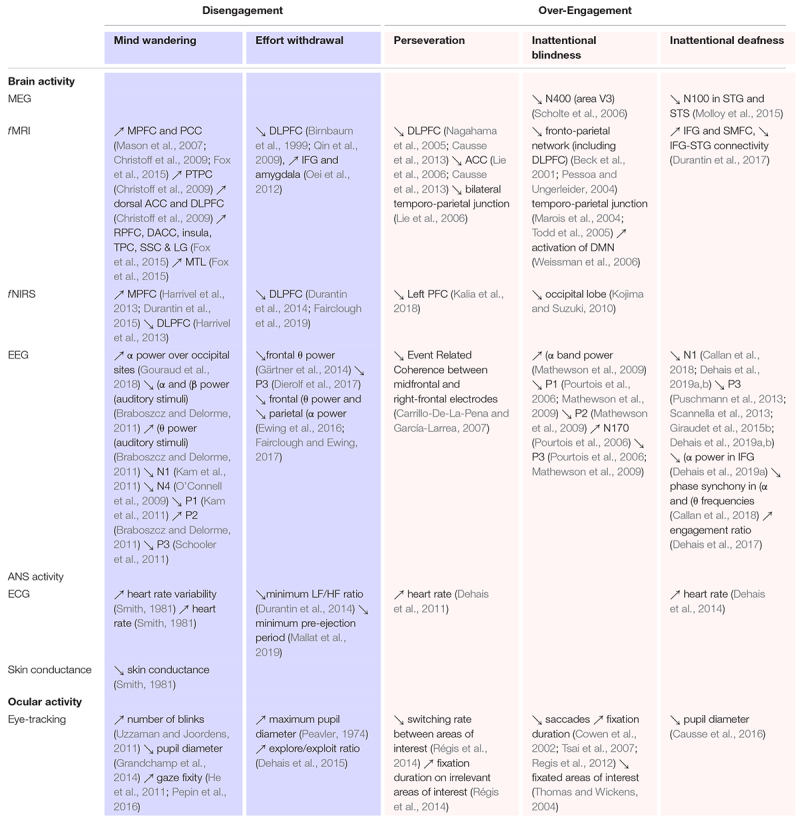

*The blue and pink color-code respectively tags states induced by low and high task demand. RIFG, right inferior frontal gyrus; DMN, default mode network, MFG, middle frontal gyrus; ACC, anterior cingulate cortex; LFC, lateral frontal cortex; STC, superior temporal cortex; PFC, prefrontal cortex; PCC, posterior cingulate cortex; MPFC, medial prefrontal cortex; PTPC, posterior temporoparietal cortex; DLPFC, dorsolateral prefrontal cortex; RPFC, rostrolateral prefrontal cortex; DACC, dorsal anterior cingulate cortex; TPC, temporopolar cortex; SSC, secondary somatosensory cortex; LG, lingual gyrus; MTL, medial temporal lobe; SMFC, superior medial frontal cortex; IFG, inferior frontal gyrus; STS, superior temporal sulcus, STG, superior temporal gyrus.*

Secondly, attentional states, such as inattentional deafness and blindness, result from the activation of an attentional network involving the inferior frontal gyrus, the insula and the superior medial frontal cortex (Tombu et al., 2011; Callan et al., 2018; Dehais et al., 2019). These regions represent potential candidates upon which to identify attentional failures that can be complemented by monitoring dedicated primary perceptual (see Hutchinson, 2019, for a review) and integrative cortices (Molloy et al., 2015), as well as performing connectivity analyses (Callan et al., 2018). In addition, inattentional phenomena may result from the suppression of activity in the right temporo-parietal junction (TPJ), a part of the ventral network, which also blocks reorientation of attention and the processing of unexpected stimuli (Marois et al., 2004; Todd et al., 2005).

Thirdly, measures of arousal are used to characterize high engagement and delineate distinct mental states within the category of low task engagement (Figure 2). Heart rate (HR) and heart rate variability (HRV) can be used to assess the activation or co-activation of the two branches of the autonomous nervous system (i.e., sympathetic or parasympathetic) (Fairclough, 2008; Qin et al., 2009; Kreibig, 2010). For instance, fluctuations in HR are commonly observed during high task engagement and high arousal (De Rivecourt et al., 2008; Qin et al., 2009; Dehais et al., 2011). Moreover, spectral analyses computed over the EEG signal revealed that shifts in parietal alpha [8–12] Hz and frontal theta [4–8] Hz are relevant markers of arousal (see Borghini et al., 2014, for a review, Senoussi et al., 2017).

Finally, behavioral metrics such as ocular behavior can complement the detection of low and high levels of engagement (Table 1). Hence, eye tracking metrics (e.g., fixation and dwell times, saccadic activity, blink rate) can be used to characterize mind wandering (He et al., 2011; Pepin et al., 2016), inattentional blindness (Thomas and Wickens, 2004; Wickens, 2005), perseveration (Régis et al., 2014), focal vs. diffused attention (Goldberg and Kotval, 1999; Regis et al., 2012; Dehais et al., 2015), and to characterize the level of attentional engagement in a visual task (Cowen et al., 2002; Tsai et al., 2007).

These metrics provide some relevant prospects to identify the targeted deleterious mental states for especially for field studies as long as portable devices are concerned. It is worth noting that the extraction of several features (e.g., time and frequency domains) and the use of several devices is a way for robust diagnosis. Moreover, contextual information (e.g., time of the day, time on task) should be considered as well as actions on the user interface and system parameters (e.g., flight parameters) if available so as to better quantify the user’s mental state.

## Solutions to Mitigate Degraded Performance

This review has identified some undesired mental states that account for degraded performance (see section “Understanding Performance Related Mental States” and “Solutions to Mitigate Degraded Performance”). A crucial step is to design cognitive countermeasures to prevent the occurrence of these phenomena. The formal framework that we proposed (see Table 1) paves the way to design neuro-adaptive technology for augmented cognition and enhanced human-machine teaming (Peysakhovich et al., 2018; Krol et al., 2019; Stamp et al., 2019). The implementation of such neuro-adaptive technology relies on a pipeline that consists of a signal acquisition step, a preprocessing step to improve the signal-to-noise ratio, a feature extraction step, a classification step to diagnose the current mental states, and lastly an adaptation step (Zander and Kothe, 2011; Roy and Frey, 2016). This last step implies the implementation of formal decisional unit (Gateau et al., 2018) that dynamically close the loop by triggering the most appropriate cognitive countermeasures (May and Baldwin, 2009). There are currently three types of mitigating solutions to instigate a change in behaviors via: (1) adaptation of the user interface, (2) adaptation of the task and of the level automation, and the (3) “neuro-adaptation” of the end-users.

### Adaptation of the User Interface

The first category of neuroadaptive countermeasure consists of triggering new types of notifications via the user interface to alert of impeding hazards. The design of these countermeasures is generally grounded on neuroergonomics basis so that these warning can reach awareness when other means have failed. Following this perspective, Dehais et al. (2010, 2012), Imbert et al. (2014) and Saint Lot et al. (2020) have demonstrated that very brief (∼200 *ms*) and located information removal was an efficient mean to mitigate perseveration by forcing disengagement from non-relevant tasks. Souza et al. (2016) demonstrated that digital nudging (see Weinmann et al., 2016) could be used to mitigate poor decision making and cognitive bias associated with perseveration. Imbert et al. (2014) designed attention-grabbing stimuli grounded on vision research and demonstrated that yellow chevrons pulsing at a cycle of 1 Hz can re-orientate attention and mitigate inattentional blindness. Jahanpour et al. (2018) has explored the design of pop-up videos that display the gestures to be performed by exploiting the property of mirror neurons. This visual “motor cue” approach was tested and drastically reduced reaction time to alerts during complex situations and appears to be a promising method to prevent effort withdrawal (Causse et al., 2012). In a similar fashion, Navarro et al. (2010) implemented a force-feedback steering wheel to prime the motor response from the driver. This device was found to optimize drivers’ behavior during demanding driving scenario. This latter study demonstrated how tactile notifications can alert human operators of impeding hazards (Lewis et al., 2014; Russell et al., 2016), especially when other sensory channels of information (e.g., visual stream) are saturated (Elliott et al., 2011). However, there are potential limits to the effectiveness of these types of notifications and stimulation (Murphy and Dalton, 2016; Riggs and Sarter, 2019). Other research indicates that multimodal alerts (Giraudet et al., 2015a; Gaspar et al., 2017) increase the likelihood of attentional capture. In addition, Lee et al. (2018) designed a motion seat that modifies the driver’s seat position and posture across time to diminish the potential deleterious effect of mind wandering. Similar concepts have been applied to aviation (Zaneboni and Saint-Jalmes, 2016).

### Task and Automation Adaptation

The second category of neuroadaptive countermeasure is the dynamic reallocation of tasks between humans and automation to maintain the performance efficacy of the operators (Freeman et al., 1999; Parasuraman et al., 1999; Prinzel et al., 2000; Scerbo, 2008; Stephens et al., 2018). The underlying concept in this case is to optimize human-human or human(s)-system(s) cooperation according to criteria of availability and skills of human and artificial agents (Gateau et al., 2016). For instance, Prinzel et al. (2000) utilized the continuous monitoring of brain waves that could be used to drive the level of automation and optimize the user’s level of task engagement. Similarly, some authors managed to optimize air traffic controllers’ task demand by triggering different levels of assistance (Aricò et al., 2016; Di Flumeri et al., 2019). These latter studies reported better human performance when neuro-adaptive automation was switched on compared to other conditions. Gateau et al. (2016) implemented an online attentional state estimator coupled with a stochastic decision framework to dynamically adapt authority sharing between human and robots in a search and rescue scenario to prevent effort withdrawal on the part of the human. In a more extreme fashion, Callan et al. (2016) revealed that it is possible to decode user motor intention so automation can perform on behalf of the user to drastically reduce the response time in emergency situations (e.g., collision with terrain). In the future, it is assumed that aircraft designers will implement adaptive automation technology that takes over from the pilots by either inhibiting their inputs on the flight deck or performing automated evasive actions (e.g., automatic pull-up) to prevent from perseveration. A complementary approach is to modulate task difficulty to maintain the task challenging but achievable while preventing the occurrence of task withdrawal (Ewing et al., 2016) or mind wandering (Freeman et al., 2004; Ewing et al., 2016). The online modulation of the tasks does not necessarily reduce the difficulty of the task. For instance, Verwey and colleagues demonstrated that the addition of an entertaining task while driving improved the operator’s ability to maintain their level of task engagement over long period of time (Verwey and Zaidel, 1999). Similarly, it has been suggested that switching the types of tasks presented to the user can prevent the deleterious effect of fatigue and disengagement (Hockey, 2011).

### Neuro-Adaptation of the End-User(s)

The third and final category aims to warn the users of their mental state and “stimulate” neurological activity in order to augment performance. One of the most promising approach relies on the implementation of Neurofeedback (see Gruzelier, 2014; Enriquez-Geppert et al., 2017 for reviews). The principle of the latter technique is to provide feedback in real-time to the users of their mental states in the form of a visual, tactile or auditory stimulus. The users can utilize these signals learn to regulate their brain activity and in return improve their executive (Enriquez-Geppert et al., 2013), mental flexibility (Enriquez-Geppert et al., 2014), and attentional abilities (Egner and Gruzelier, 2001) as well as enhance their task engagement (Egner and Gruzelier, 2004). However, the effects of this approach on mind wandering remain unclear (Gonçalves et al., 2018). Transcranial direct current stimulation (tDCS) represents a technique of neuromodulation that can be used to boost executive functioning (see Callan and Perrey, 2019; Cinel et al., 2019). This portable device can be combined with EEG and fNIRS and used in the context of real-life task performance for the purpose of on-line neuromodulation (McKendrick et al., 2015; Gateau et al., 2018). For example, a number of studies support the position that neurostimulation can: enhance mental flexibility and mitigate perseveration (Leite et al., 2011; Jeon and Han, 2012), improve visual attention (Falcone et al., 2012; Nelson et al., 2015), improve executive functioning in multitasking situations (Nelson et al., 2016) and increase alertness (McIntire et al., 2014; Nelson et al., 2014). There are other types of environmental stimulation such as vivid light exposure, especially during night flights, which can promote an optimal level of alertness (see Anund et al., 2015) without altering flight crew performance (see Caldwell et al., 2009). Promising results have also been highlighted by using light exposure in cars (Taillard et al., 2012). The use of light exposure and tDCS should be considered with caution as there is a need to investigate the very long-term efficiency and potential side effects. Alternatively, some authors proposed to use cold-air jet to decrease hypovigilance (Reyner and Horne, 1998), but with contradictory findings.

### Synthesis of Neuro-Adaptive Solutions

**FIGURE 4:**
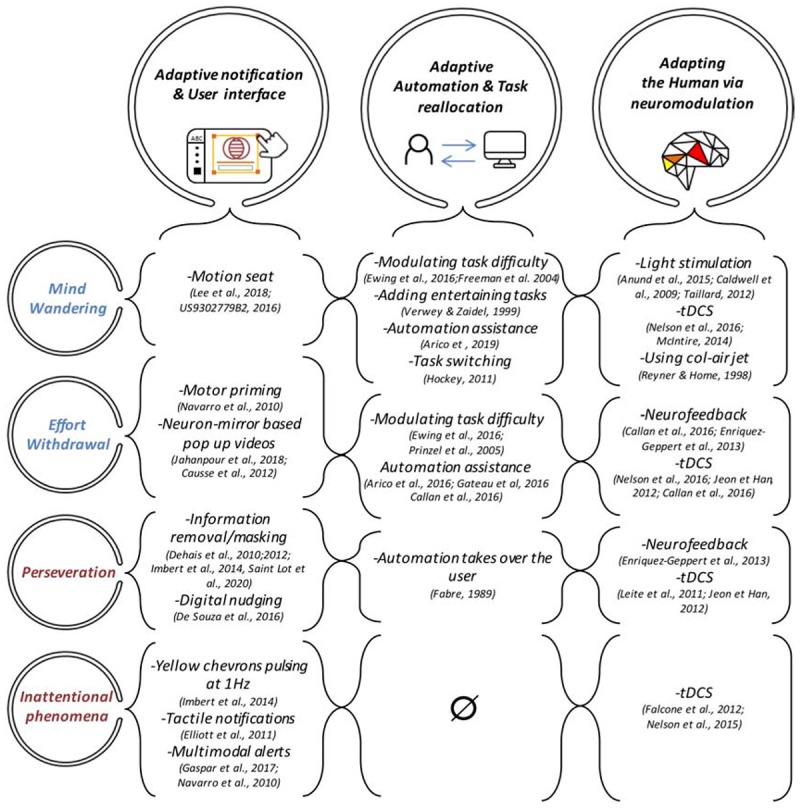
The three types of Neuroadaptive countermeasures dedicated to mitigate the undesirable mental states. Inattentional deafness and Inattentional blindness mental states were merged into “Inattentional phenomena” as no neuroadaptive countermeasure were implemented to explicitly address failure of auditory attention to the exception of multimodal alerts. Moreover, no adaptive automation-based solutions were designed to prevent from inattentional states. This demonstrates the need to conduct more research in this direction.

The following illustration (see Figure 4) depicts the three families of neuro-adaptive based solutions to mitigate performance impairment.

The three types of neuroadaptive solutions offer promising prospects to mitigate the onset and likelihood of undesirable neurocognitive states. However, they should be delivered in a transparent, meaningful, and timely manner (i.e., when needed) so they are relevant and understood (Dorneich et al., 2016; Sebok et al., 2017), otherwise these types of intervention have the potential for undesirable consequences, such as performance impairment and reduced trust in technology; this point is particularly true for adaptive automation solutions that take over from humans, especially under critical scenarios (see Dorneich et al., 2016; Dehais et al., 2019). One solution is to combine different families of neuroadaptive cognitive countermeasures to maximize their efficiency. Ideally, we would argue to use a gradient of solutions such as (1) the continuous display of the users’ mental states via neurofeedback techniques to give them the opportunity to regulate their brain activity; (2) using notifications to suggest to the users to delegate some tasks to automation in case they don’t manage to modulate their mental states; (3) adapting the user interface (e.g., information removal, flashing yellow chevrons) in case of a critical situation is detected and the previous solutions were inefficient; and (4) taking over if the users do not respond to any of the previous countermeasures.

## Conclusion

This paper has argued that the concept of a limited resource provides a limited explanation for the breakdown of operational performance. Our neurophysiological analysis describes a number of additional mechanisms, such as perseveration and effort withdrawal, which do not represent finite resources *per se*. In both cases, explanations for performance breakdown are based upon neurological processes, such as dominance of specific neural networks or the heightened activity of specific mechanisms. We propose a two-dimensional framework of engagement and arousal that captures the importance of specific degraded mental sates associated with poor performance. The rationale for including the transactional concept of engagement in this scheme is to account for the goal-oriented aspect of cognition. The benefit of including the transactional concept of arousal is to make a distinction between two categories of disengagement, one that is accompanied by high arousal (effort withdrawal) and low arousal (mind wandering) – and to link this conceptual distinction to known neurophysiological effects (see Figure 1). Nonetheless, this approach remains at the conceptual level and minimizes connections to the complexity of brain functioning. To that end, we reviewed and identified several markers at the neurophysiological, physiological and behavioral level of undesirable mental states linked to poor performance.

This neuroergonomic framework encompasses operationali- zations of these undesirable states that can be monitored continuously in an objective fashion. Such considerations eventually lead to propose a typology of neuroadaptive countermeasures and open promising perspectives to mitigate the degradation of human performance. However, to the authors’ very best knowledge, most of the neuroadaptive experimental studies have focused on human-machine dyad situations. We believe that recent research on hyperscanning (Babiloni and Astolfi, 2014), physiological synchrony (Palumbo et al., 2017) and collaborative BCIs (Cinel et al., 2019) have opened promising prospects to improve teaming such as human-human, human(s)-machine(s) interactions. Future research should involve more complex teaming scenarios and enrich the different neuroadaptive solutions. We sincerely hope that this review will encourage research efforts to identify additional degraded mental states and associated neurophysiological markers as well as to implement neuroadaptive solutions for safer and efficient human-human and human(s)-machine(s) interactions.

## Author Contributions

All authors have made a substantial and intellectual contribution to this review.

## Conflict of Interest

The authors declare that the research was conducted in the absence of any commercial or financial relationships that could be construed as a potential conflict of interest.
